# The predictive value of serum inflammatory markers for the severity of cervical lesions

**DOI:** 10.1186/s12885-024-12561-7

**Published:** 2024-06-28

**Authors:** Lin Qin, Lina Zhang

**Affiliations:** Senior Department of Obstetrics & Gynecology, the Seventh Medical Center of PLA General Hospital, Beijing, China

**Keywords:** Colposcopy, Cervical lesions, NLR, PLR, MLR, SII

## Abstract

**Objective:**

Exploring the predictive value of NLR, PLR, MLR, and SII for the severity of cervical cancer screening abnormalities in patients.

**Methods:**

A retrospective analysis was conducted on the data of 324 patients suspected of cervical lesions due to abnormal TCT and/or HPV in our hospital from January 2023 to December 2023, who underwent colposcopy. The pathological results of colposcopic biopsy confirmed that there were 140 cases of chronic cervicitis, which classified as the group without cervical lesions. The cervical lesion group included 184 cases, including 91 cases of LSIL, 71 cases of HSIL, and 22 cases of cervical cancer. Compared the differences in preoperative peripheral blood NLR, PLR, MLR, and SII among different groups of patients, and evaluated their predictive value for the severity of cervical lesions using Receiver Operating Characteristic (ROC) curves.

**Results:**

The levels of NLR, PLR, and SII in the group without cervical lesions were lower than those in the group with cervical lesions (*p* < 0.05), and there was no statistically significant difference in MLR (*p* > 0.05). The comparison of NLR among LSIL, HSIL, and cervical cancer groups showed statistically significant differences (*p* < 0.05), while PLR, MLR, and SII showed no statistically significant differences (*p* > 0.05). The AUC of peripheral blood NLR, PLR, and SII for predicting cervical lesions were 0.569, 0.582, and 0.572, respectively. The optimal cutoff values were 2.3,176.48, and 603.56. The sensitivity and specificity were 38.6% and 73.6%, 28.8% and 85.7%, 37.5% and 76.4%, respectively. At the same time, the joint testing of the three had the highest efficiency, with sensitivity of 69% and specificity of 45%.

**Conclusion:**

Although the peripheral blood NLR, PLR, and SII of the cervical lesions patients were higher than those without cervical lesions in cervical cancer screening abnormal patients, the predictive ROC curve discrimination was low. Therefore, it is not recommended to use preoperative peripheral blood inflammatory markers as markers for cervical cancer screening abnormal patient diversion.

## Introduction

Cervical cancer is a malignant tumor of the female reproductive system, ranking fourth among all malignant tumors [1], which posing a threat to women’s health and life. The popularization of cervical cancer screening has improved the diagnostic rate of cervical intraepithelial neoplasia (CIN) and reduced the incidence of advanced cervical cancer. At the same time, the global goal is to eliminate cervical cancer through three-level prevention [2] measures [3–5]. Therefore, early diagnosis of CIN has become an important measure for the prevention and treatment of cervical cancer. In clinical practice, patients with abnormal for cervical cancer screening need to be referred for colposcopy and biopsy, which increases the detection rate of cervical precancerous lesions and cervical cancer. However, the false positive rate and colposcopy referral rate both increase, and excessive intervention leads to an increase in complications [6]. Previous studies had confirmed that the levels of neutrophils, platelets, and lymphocytes in peripheral blood could cause systemic immune inflammatory responses. Neutrophil/lymphocyte ratio (NLR), lymphocyte/monocyte ratio (LMR), and platelet/lymphocyte ratio (PLR) were three non-specific indicators of systemic inflammation [7, 8], which had been proven to predict the prognosis of multiple malignant tumors [9–11]. The Systemic Immune-inflammation Index (SII) was a new inflammatory marker, calculated using the formula Platelet count × Neutrophil count/Lymphocyte count was now also used as an important biomarker for prognostic evaluation of various cancer patients [12–17]. Neutrophils are a major subgroup of white blood cells that promote tumor cell proliferation, angiogenesis, and metastasis by producing pro angiogenic chemokines, cytokines, and growth factors such as vascular endothelial growth factor. Monocytes can release various chemokines and cytokines, promoting tumor progression and metastasis [18], while lymphocytes produce cytokines that inhibit tumor cell proliferation, metastasis, or promote cell death [19].Platelets can stimulate the proliferation and adhesion of tumor cells to other cells by secreting cytokines, chemokines, etc., and help tumor cells evade immune monitoring, promoting the growth and spread of tumor cells [20–21]. Research has shown that elevated levels of neutrophils, monocytes, and platelets in peripheral blood, as well as lower levels of lymphocytes, may correspond to higher invasiveness of tumors [22]. Some inflammatory factors in the blood were related to the occurrence and development of tumors, and had been reported in lung cancer [23], breast cancer [24], and bladder cancer [25]. Research showed that chronic inflammation may play an important role in the development of breast cancer, and had a certain impact on the occurrence, development, diagnosis and prognosis of breast cancer [26–29]. HU et al. [30] demonstrated that an increase in PLR values indicated a higher likelihood of inguinal lymph node metastasis in patients with penile cancer. ZHU et al. [31] found that there was a statistically significant difference in NLR between lung cancer patients and healthy individuals, with lung cancer patients having a higher NLR, the NLR cutoff value was 2.14. An increase in NLR was closely related to an increased risk of lung cancer, and this correlation was more pronounced within one year before the diagnosis of lung cancer [32, 33]. Research had found that the NLR of cervical cancer patients was significantly higher than that of the healthy population group. ROC curve analysis showed that NLR was a predictive factor for the diagnosis of cervical cancer, with an optimal critical value of 2.02 (AUC = 0.682, sensitivity of 71%, specificity of 60%) [34]. However, there was no clear conclusion on the use of preoperative serum inflammatory markers in the cervical cancer screening abnormal patients. In view of this, this study retrospectively analyzed 324 patients with cervical cancer screening abnormal who underwent colposcopy as the research subjects, with biopsy pathology results as the gold standard for diagnosis. Attempting to find more effective alternative biomarkers for predicting cervical lesions, providing more accurate indications for colposcopy, in order to achieve precise patient diversion, reduce unnecessary colposcopy referrals, allocate medical resources reasonably, improve screening efficiency, and reduce overtreatment.

## Materials and methods

### The general materials

Retrospective analysis of data from patients with suspected cervical lesions due to Thin Cytology Test (TCT) and/or Human Papilloma Virus (HPV) abnormal who underwent colposcopy and biopsy in our hospital from January 2023 to December 2023. Inclusion criteria: Patients with cervical cancer screening abnormal who underwent colposcopy in our hospital, and tissue biopsy would be performed simultaneously during procedure; The clinical and pathological data were all based on the results of our hospital; Non pregnant women; No history of treatment for cervical lesions. Exclusion criteria: Patients with major organ failure such as heart, liver, and kidneys; Patients who received antibiotic treatment before enrollment; Patients with obvious infections; Combination of malignant tumor, blood system or other infectious diseases; Those who were dissatisfied with colposcopy and affect the diagnostic results; oral medication that affected inflammatory markers(e.g., steroid); Incomplete clinical data. A total of 324 patients were enrolled, aged 18–77 years, with a median age of 47 (36,55) years. BMI fluctuates between 16.2 and 35.34, with a median BMI of 24 (22.5,25.78). This study was approved by ethics committees of Senior Department of Obstetrics & Gynecology of PLA General Hospital having approval no. 2024KY007-KS001 on January 19, 2024.

### Methods

Extract general information such as age of enrolled patients and routine blood test within one week before colposcopy from electronic medical records, and calculate NLR, PLR, MLR, and SII.

### Statistical analysis

SPSS 23.0 software was used for statistical analysis. The measurement data that do not conform to the normal distribution, the median and quartile were used, and the Mann-Whitney U test was used for inter-group comparison. Compare three groups and applied Kruskal-Wallis test. The predictive value was evaluated using the ROC curve analysis. The difference was statistically significant (*p* < 0.05).

## Results

A retrospective analysis was conducted on the clinical and pathological data of 324 patients enrolled, and the pathological results of colposcopic biopsy confirmed that there were 140 cases (43.2%, 140/324) of chronic cervicitis in the group without cervical lesions. And the cervical lesion group consisted of 184 cases (56.8%, 184/324), including 91 cases of low-grade squamous intraepithelial lesion(LSIL) (28%, 91/324), 71 cases of high-grade squamous intraepithelial lesion (HSIL) (21.9%, 71/324), and 22 cases of cervical cancer (6.7%, 22/324).

### Comparison of onset age, NLR, PLR, MLR, and SII between the group without cervical lesions and the group with cervical lesions

The onset age, NLR, PLR, and SII levels of the group without cervical lesions were significantly different from those of the group with cervical lesions (*p* < 0.05), indicating that the onset age of the group without cervical lesions was higher than that of the group with cervical lesions, while the levels of NLR, PLR, and SII were lower than those of the latter. However, there was no statistically significant difference in BMI, MLR between the two groups (*p* > 0.05), as shown in Table 1.

**Table 1:** Comparison of clinical data and inflammatory indicators between two groups

Index	the group without cervical lesions(*n* = 140)	the group with cervical lesions(*n* = 184)	Z value	*P* value
Onset age(years)	48(39.25,56)	43(35,54)	-2.405	0.016
BMI	23.7(22.12, 26.24)	24.17(22.79, 25.63)	-1.704	0.088
NLR	1.81(1.33,2.37)	1.99(1.51, 2.59)	-2.124	0.034
PLR	130.01(108.06,162.4)	142.68(116.3,179.32)	-2.518	0.012
MLR	0.16(0.13,0.20)	0.17(0.13,0.20)	-1.124	0.261
SII	468.5(322.83,602.32)	497.95(356.84,717.08)	-2.217	0.027

### Comparison of onset age, NLR, PLR, MLR, and SII among LSIL, HSIL, and cervical cancer groups in the cervical lesion group

The onset age and NLR of LSIL, HSIL, and cervical cancer groups showed statistically significant differences (*p* < 0.05), while PLR, MLR, and SII showed no statistically significant differences (*p* > 0.05) among the three groups, as shown in Table 2.

**Table 2:** Comparison of clinical data and inflammatory indicators among three groups

Index	LSIL group(*n* = 91)	HSIL group(*n* = 71)	Cervical cancer group (*n* = 22)	χ2 value	*P* value
Onset age(years)	42(35,53)	42(33,54)	54(42.75,58)	8.01	0.018
NLR	1.94(1.53,2.53)	2.20(1.56,2.85)	1.70(1.23,2.32)	6.72	0.035
PLR	140.22(116.19,178.66)	148.19(122.33,191.43)	126.5(103.48,171.06)	3.62	0.164
MLR	0.17(0.14,0.20)	0.17(0.13,0.21)	0.15(0.11,0.19)	2.69	0.261
SII	494.69(371.29,671.83)	547.66(368.79,773.11)	405.89(268.9,613.58)	4.81	0.090

### The pairwise comparison of onset age in LSIL, HSIL, and cervical cancer groups

**Fig. 1:**
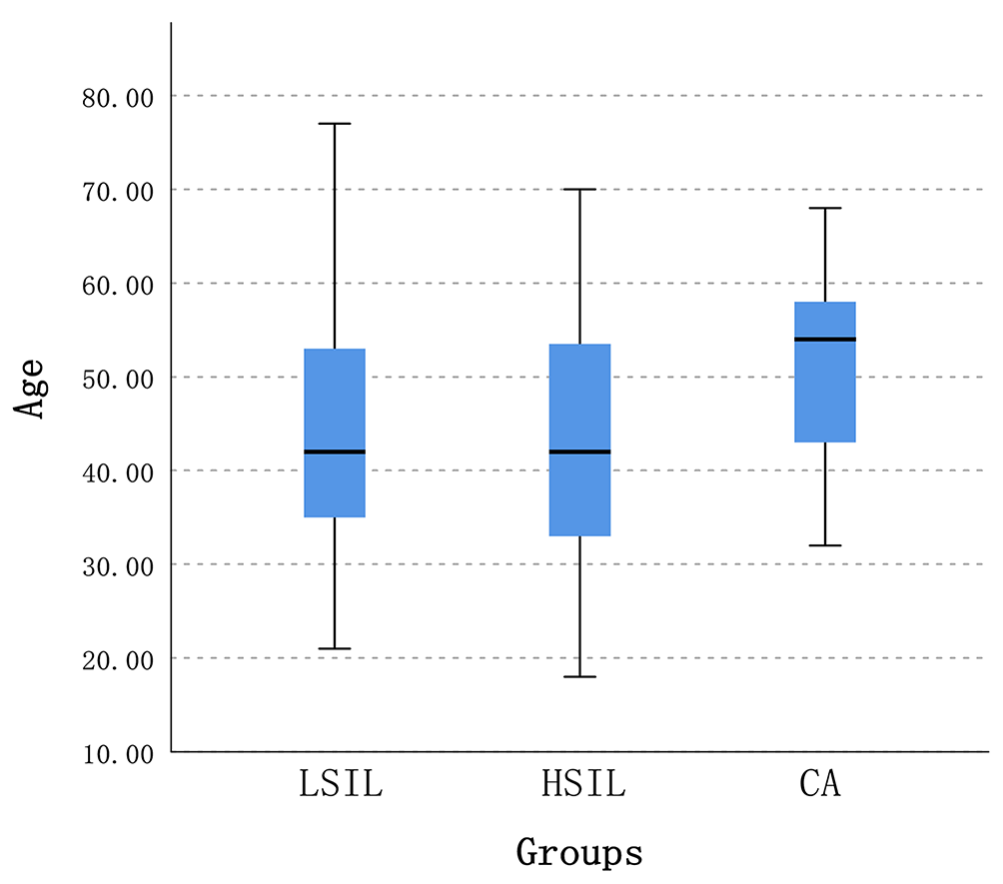
Comparison of onset age groups for LSIL, HSIL, and cervical cancer

The results showed that there was a statistically significant difference in LSIL-CA groups (*p* = 0.038) and HSIL-CA groups (*p* = 0.16), while there was no statistically significant difference in LSIL-HSIL groups (*p* > 0.05), as shown in Fig. 1.

### The pairwise comparison of NLR in LSIL, HSIL, and cervical cancer groups

**Fig. 2:**
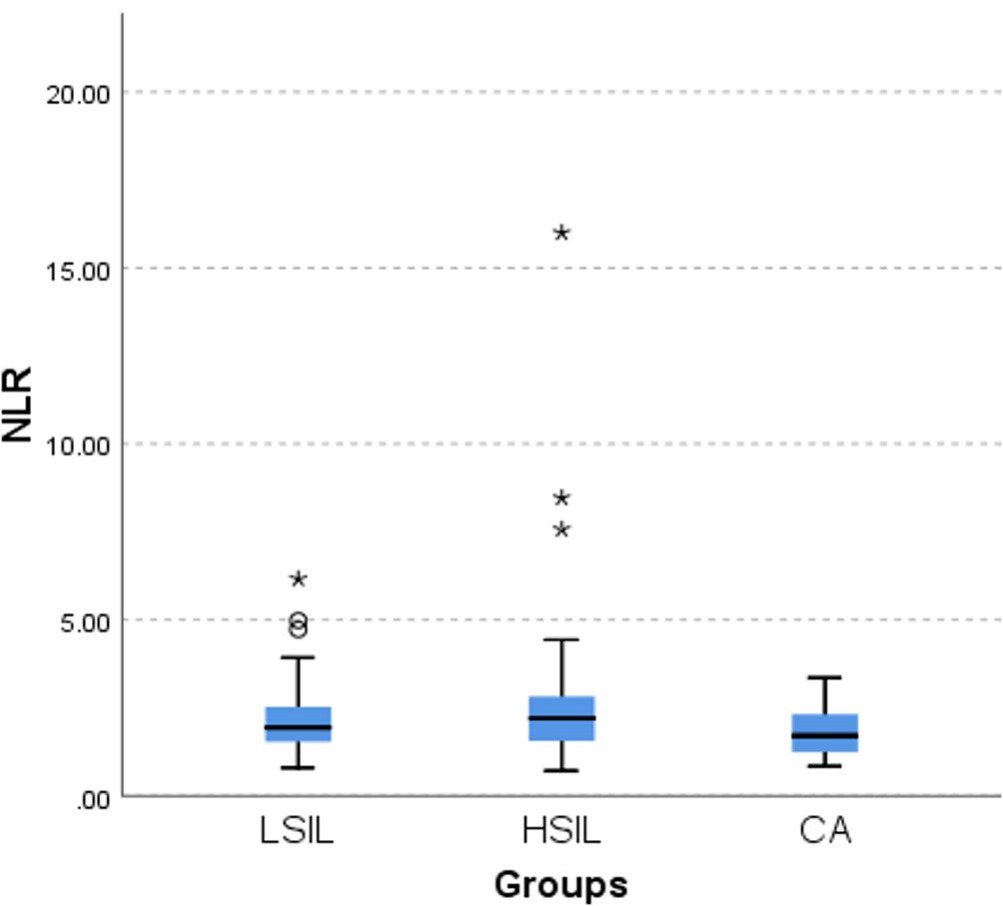
Comparison of NLR among LSIL, HSIL, and cervical cancer groups

The results showed a statistically significant difference between HSIL-CA (*p* = 0.034), while there was no statistically significant difference in LSIL-HSIL groups (*p* = 0.454) and LSIL-CA groups (*p* = 0.3) (*p* > 0.05), as shown in Fig. 2.

### The predictive value of serum inflammatory markers for cervical lesions

**Fig. 3:**
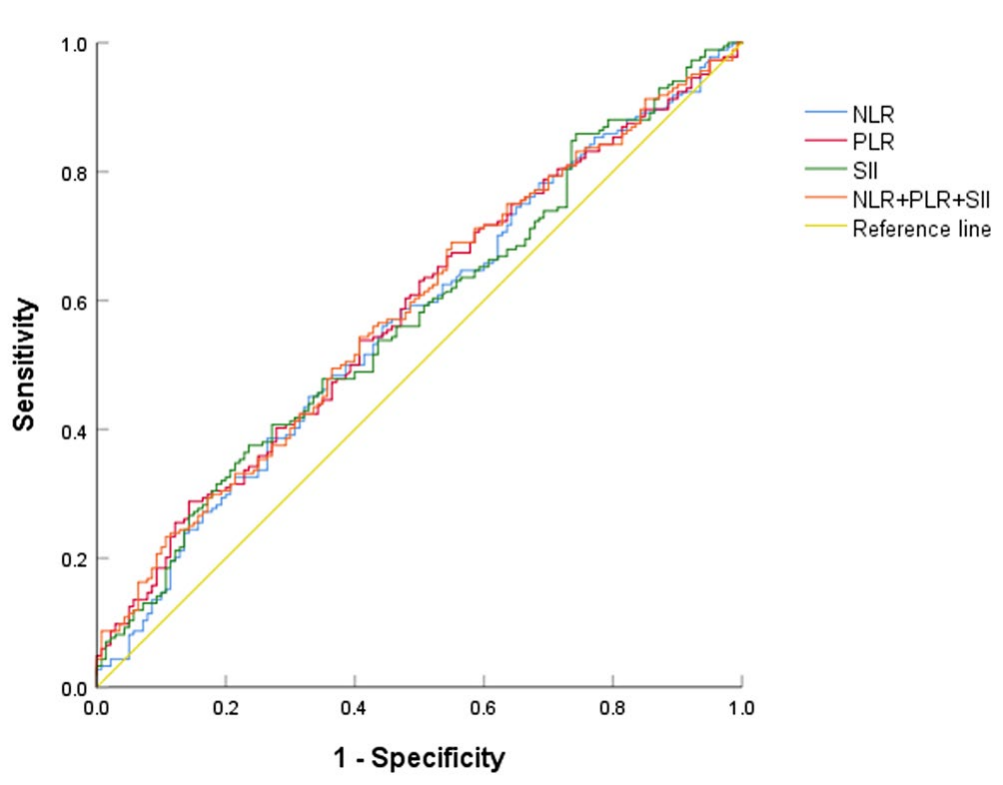
ROC curve for predicting cervical lesions using NLR, PLR, SII, and their combined detection

Using cervical lesions (LSIL, HSIL, and cervical cancer) as state variables and NLR, PLR, and SII as test variables, ROC curves were plotted. The results showed that NLR, PLR, and SII predicted AUC of cervical lesions at 0.569, 0.582, and 0.572, respectively, with the best cutoff values of 2.3,176.48,603.56. At the same time, the joint testing of the three had the highest efficiency, with a sensitivity of 69% and a specificity of 45%. Shown as Fig. 3; Table 3.

**Table 3:** Predictive value of NLR, PLR, SII, and their combined detection in predicting cervical lesions

Index	AUC	Cut-off value	Sensitivity(%)	Specificity(%)	95%CI	*P* value
NLR	0.569	2.30	38.6	73.6	0.506–0.632	0.034
PLR	0.582	176.48	28.8	85.7	0.520–0.644	0.012
SII	0.572	603.56	37.5	76.4	0.509–0.634	0.027
NLR + PLR + SII	0.584	/	69	45	0.522–0.646	0.010

## Discussion

Cervical cancer is a common gynecological tumor in women [20, 21], and is also the main cause of cancer-related deaths for women in developing countries [35]. Human papilloma virus (HPV) is the primary cause of cervical dysplasia and cervical cancer [36]. Cervical cancer is a complex process from precancerous lesions to cancer, and CIN reflects the continuous process of cervical cancer occurrence and development. Screening to detect CIN and timely treatment of HSIL is an effective measure to prevent cervical cancer. With the popularization of cervical cancer screening, the early diagnosis rate of cervical cancer has significantly improved, and the survival rate of patients has been greatly improved [37]. The purpose of cervical cancer screening is to identify patients with precancerous lesions of the cervix. Those with abnormal screening results should undergo colposcopy or cervical biopsy. If diagnosed with HSIL, the cervical lesion tissue should be removed to prevent progression to cervical cancer. In this study, patients with abnormal for cervical cancer screening who underwent colposcopy were ultimately confirmed by pathology to have HSIL accounting for 21.9% and cervical cancer accounting for 6.7%. The sensitivity of colposcopy in diagnosing HSIL and above was relatively low. Therefore, this study aimed to search for suitable serum inflammatory markers to improve the efficacy of colposcopy in patients with cervical cancer screening abnormal.

The results of this study showed that in patients with cervical cancer screening abnormal, the levels of NLR, PLR, and SII in the group without cervical lesions were lower than those in the group with cervical lesions. However, if peripheral blood inflammatory markers were used as biomarkers for cervical cancer screening abnormal patients, their area under the ROC curve for predicting cervical lesions also needed to be considered. The AUC of peripheral blood NLR, PLR, SII, and their combined prediction for cervical lesions were 0.569, 0.582, 0.572, and 0.584, respectively, all < 0.6, indicating that the predictive value of serum inflammatory markers for cervical lesions was not reliable, and the conclusion was difficult to extrapolate. According to the above analysis, NLR, PLR, and SII had low predictive discrimination for patients with cervical cancer screening abnormal, and were influenced by various factors such as systemic inflammatory response, making them unstable. Therefore, in the absence of clear mechanisms related to tumor immunity, the author did not recommend using NLR, PLR, and SII as diversion markers for the patients with cervical cancer screening abnormal.

Research had confirmed that chronic inflammation plays an important role in the occurrence and development of cervical cancer [38, 39], and terminal malignant tumors were accompanied by neutropenia and lymphocyte depletion, and thrombocytosis [40]. Taguchi et al. [41] showed that high levels of NLR were effective predictors of recurrence and metastasis in cervical cancer patients after treatment. Trinh et al. [42] showed that an increased in NLR and PLR before treatment resulted in a lower PFS and OS for cervical cancer after radiotherapy; and LMR increased before treatment had a good prognosis. SII was a new inflammatory biomarker that includes three types of peripheral blood inflammation biomarkers and was considered superior to other inflammatory indicators [43]. The prognostic value of SII in patients with stage I-II esophageal squamous cell carcinoma was superior to NLR, PLR, and MLR [44]. Meanwhile, studies had shown that high pre-treatment SII in cervical cancer patients indicated low OS and PFS [45]. Tas et al. [34] showed that peripheral blood NLR and PLR could distinguish cervical cancer from precancerous lesions, and the NLR and PLR of cervical cancer patients were higher than CIN. Another study also confirmed that the PLR of the cervical cancer group was significantly increased compared to the low-grade squamous intraepithelial lesions of the cervix, the highly squamous intraepithelial lesions of the cervix, and the healthy control group [46]. Preoperative NLR categorization was a strong independent prognostic factor for recurrences after surgical excision of CIN2, CIN3, and carcinoma in situ, the odd ratio for recurrence significantly higher in patients with NLR ≥ 2 (5.2% and 27.3%) [47]. When NLR >1.9, the patient with cervical intraepithelial neoplasia has a high recurrence rate, and they need excision more tissues during excisional surgery [48]. Xu et al. [49] showed that a high NLR value independently predicted CIN and the stage of CIN. At present, there were many reports on the use of inflammatory markers in peripheral blood for the diagnosis and prognosis of cervical cancer [41, 50, 51], but no relevant literature reports on the diversion of patients with cervical cancer screening abnormal have been found yet.

In addition, this study had the following limitations: firstly, as a single center retrospective study, there were differences in onset age, BMI, and parity among the patients in different groups, which may caused some confounding bias, and preoperative single routine blood test may produced random errors. Secondly, cervical lesions are associated with HPV infection [21], but the HPV infection status, types, and duration among different groups of patients were not included in the research, which may caused some bias in the results. Finally, this study did not follow up the object of study, which had certain limitations on the research results. In the future studies, further follow-up is needed to determine the changes in subsequent detection indicators to confirm the clinical application value of each detection scheme.

## Conclusion

In summary, although the peripheral blood NLR, PLR, and SII of cervical lesions patients are higher than those without cervical lesions in cervical cancer screening abnormal patients. Based on current data analysis, the significance of preoperative peripheral blood inflammatory markers in predicting cervical lesions is not clear. Therefore, it is not recommended to use preoperative peripheral blood inflammatory markers as markers for the diversion of cervical cancer screening abnormal patients.

## Data Availability

The datasets used and/or analysed during the current study available from the corresponding author on reasonable request.
